# Novel Polymorphisms and Genetic Characteristics of the Prion Protein Gene in Pheasants

**DOI:** 10.3389/fvets.2022.935476

**Published:** 2022-07-12

**Authors:** Kyung Han Kim, Yong-Chan Kim, Byung-Hoon Jeong

**Affiliations:** ^1^Korea Zoonosis Research Institute, Jeonbuk National University, Jeonju, South Korea; ^2^Department of Bioactive Material Sciences, Institute for Molecular Biology and Genetics, Jeonbuk National University, Jeonju, South Korea

**Keywords:** pheasant, prion, prion protein gene (*PRNP*), hexapeptide, polymorphisms, single nucleotide polymorphism

## Abstract

Transmissible spongiform encephalopathies (TSEs) also known as prion diseases, are fatal neurodegenerative diseases. Prion diseases are caused by abnormal prion protein (PrP^Sc^) derived from normal prion protein (PrP^C^), which is encoded by the prion protein gene (*PRNP*). Prion diseases have been reported in several mammals. Notably, chickens, one species of bird, have not been reported to develop prion diseases and showed resistance to bovine spongiform encephalopathy (BSE) infection. However, genetic polymorphisms of the *PRNP* gene and protein structure of the prion protein (PrP) related to vulnerability to prion diseases have not been investigated in pheasants, another species of bird. We performed amplicon sequencing of the pheasant *PRNP* gene to identify genetic polymorphisms in 148 pheasants. We analyzed the genotype, allele and haplotype frequencies of the pheasant *PRNP* polymorphisms. In addition, we evaluated the effect of genetic polymorphisms of the pheasant *PRNP* gene on pheasant PrP by the AMYCO, PROVEAN, PolyPhen-2 and PANTHER softwares. Furthermore, we compared the amino acid sequences of tandem repeat domains and secondary and tertiary structures of prion proteins (PrPs) among several animals. Finally, we investigated the impact of non-synonymous single nucleotide polymorphisms (SNPs) on hydrogen bonds and tertiary structures of pheasant PrP by Swiss PDB viewer software. We identified 34 novel genetic polymorphisms of the pheasant *PRNP* gene including 8 non-synonymous SNPs and 6 insertion/deletion polymorphisms. Among the non-synonymous SNPs, the L23F, G33C and R177Q SNPs showed that they could have a deleterious effect on pheasant PrP. In addition, the R177Q SNP was predicted to show an increase in amyloid propensity and a reduction in hydrogen bonds of pheasant PrP. Among the insertion/deletion polymorphisms, c.163_180delAACCCGGGGTATCCCCAC showed that it could have a detrimental effect on pheasant PrP. Furthermore, secondary and tertiary structures of pheasant PrP were predicted to have structures similar to those of chicken PrP. To the best of our knowledge, this is the first study on genetic polymorphisms of the pheasant *PRNP* gene.

## Introduction

Transmissible spongiform encephalopathies (TSEs), also known as prion diseases, are incurable and malignant neurodegenerative disorders. TSEs are caused by misfolded abnormal prion protein (PrP^Sc^) derived from normal prion protein (PrP^C^) encoded by the prion protein gene (*PRNP*) (1). PrP^Sc^ is characterized by a higher amyloid propensity and a higher proportion of β-sheet structure compared to PrP^C^. Prion diseases have been classified as various types including Creutzfeldt-Jakob disease (CJD), Gerstmann-Sträussler-Scheinker syndrome (GSS) and fatal familial insomnia (FFI) in humans, bovine spongiform encephalopathy (BSE) in cattle, feline spongiform encephalopathy (FSE) in cats and cheetahs, transmissible mink encephalopathy in minks, scrapie in sheep and goats and chronic wasting disease (CWD) in elk and deer (2–7).

Several single nucleotide polymorphisms (SNPs) of the *PRNP* gene play a pivotal role in the conversion of PrP^C^ to PrP^Sc^ and have been related to susceptibility to prion diseases. In humans, *PRNP* codons 129 and 219 SNPs are associated with susceptibility to CJD. In particular, M129V and E219K heterozygotes confer resistance to CJD (2–4). In sheep, the susceptibility to scrapie is associated with haplotypes of *PRNP* polymorphisms at codons 136, 154 and 171. A_136_R_154_R_171_/A_136_R_154_R_171_ diplotype is strongly associated with resistance to scrapie. However, V_136_R_154_Q_171_/V_136_R_154_Q_171_ diplotype is related to susceptibility to scrapie (5, 8). In goats, 9 polymorphisms including codons T110P, G127S, M142I, G145D, N146D,S, R154H, R211Q and Q222K are associated with scrapie (5, 9–11). In addition, the M132L SNP of the *PRNP* gene in elk plays a pivotal role in the conversion of PrP^Sc^ in CWD (12–14). Conversely, representative prion disease-resistant animals, including dogs and horses have specific amino acids related to resistance in the conversion of PrP^C^ to PrP^Sc^. In dog PrP, D163 located on the α1-β2 loop provides stability to the PrP structure and prolongs the incubation period in prion-infected transgenic mice carrying this allele (15, 16). In horse PrP, S167 located on the β2-α2 loop confers structural stability of equine PrP (17–19). Thus, the genetic characteristics of the *PRNP* gene are important in the susceptibility or resistance to prion diseases.

In birds, chickens showed resistance to experimental infection of BSE (20). Amino acid sequences of the PrP were predicted to have low amyloid propensity. In addition, there were no SNPs in the open reading frame (ORF) of the *PRNP* gene in 4 breeds including Dekalb White, Ross, Ogolgye and Korean native chickens (21, 22). However, Pekin duck showed a higher amyloid propensity of PrP than chickens. Furthermore, the specific amino acids of Pekin duck PrP showed an additional two β-sheet structures compared to chicken PrP and an increase in amyloid propensity (21). Since poultry are closely related to the food industry and, prion disease is infectious, investigation of prion disease-related genetic properties is very important in several avian species.

Pheasant *(Phasianus colchicus*) is a representative domesticated bird used for eggs and meats within the family *Phasianidae*. In Korea, more than 320,000 pheasants (*Phasianus colchicus karpowi)* are raised on over 90 farms. Pheasants and chickens belong to the order Galliformes and showed close evolutionary relationships in the phylogenetic tree. However, ducks also belong to the order Anseriformes and showed a close evolutionary relationship with Galliformes (23). Thus, there is a question of whether the pheasant has chicken-like characteristics or Pekin duck-like characteristics, and it is necessary to investigate the prion disease-related characteristics of the pheasant *PRNP* gene.

In this study, we performed amplicon sequencing in the pheasant *PRNP* gene to identify genetic polymorphisms in 148 pheasants and investigated the genotype, allele and haplotype frequencies of the pheasant *PRNP* polymorphisms. In addition, we analyzed the impact of genetic polymorphisms of the pheasant *PRNP* gene on pheasant PrP using the AMYCO, PROVEAN, PolyPhen-2 and PANTHER programs. Furthermore, we compared the amino acid sequences of the tandem repeat domains of PrPs among several species. Finally, we investigated the effect of nonsynonymous SNPs on tertiary structures of pheasant PrP and compared secondary and tertiary structures of pheasant PrP with avian PrPs.

## Materials and Methods

### Ethical Statements

A total of 148 pheasants (*Phasianus colchicus*) were obtained from a slaughterhouse provided from adjacent pheasant farms in Korea. To minimize internal relationships, we randomly selected samples 3 times. All experimental protocols were approved by the Institutional Animal Care and Use Committee (IACUC) of Jeonbuk National University (JBNU 2020-209). All efforts were made to minimize the number of animals used.

### Genomic DNA Extraction

Genomic DNA was extracted from 20 mg cerebral cortex of 148 pheasants using a Bead Genomic DNA Prep kit (Biofact, Daejeon, Korea) following the manufacturer's instructions.

### Genetic Analysis

Polymerase chain reaction (PCR) was carried out to amplify the pheasant *PRNP* gene with gene-specific primers, including PRNP-F (ATAAAGGAGGTGGGGATGGG) and PRNP-R (CGTGGACACGATGTCATCTC). These primers were designed based on the pheasant *PRNP* gene (GenBank ID; 116238382). The PCR was performed using *Taq* DNA polymerase kit (Biofact, Daejeon, Korea) contained 0.2 μl of *Taq* DNA polymerase, 5 μl of 5x Band Helper, 2.5 μl of 10x *Taq* DNA polymerase buffer, 0.5 μl of 10 mM dNTP mixture, 1 μl (10 pmol) of each primer and 1 μl (50-70 ng/μl) pheasant genomic DNA and sterile deionized water in a total volume of 25 μl. The PCR conditions were followed by the manufacturer's instructions. All PCR products (917 bp) were purified using a FavorPrep gel/PCR Purification Mini Kit (FAVORGEN, Pingtung County, Taiwan). Purified PCR products were directly sequenced in both directions with an ABI 3730xl (ABI, Foster City, CA, USA). Genotyping was performed using Finch TV software (Geospiza Inc., Seattle, WA, USA).

### Statistical Analysis

Genotype, allele and haplotype frequencies were calculated by SAS 9.4 software (SAS Institute Inc., Cary, NC, USA). Linkage disequilibrium (LD), Hardy-Weinberg equilibrium (HWE) and haplotype analyses were performed by Haploview version 4.2 (Broad Institute, Cambridge, MA, USA).

### *In silico* Analysis

PROVEAN (http://provean.jcvi.org/index.php), AMYCO (http://bioinf.uab.es/amycov04/index_CompSeq.html), PolyPhen-2 (http://genetics.bwh.harvard.edu/pph2/) and PANTHER (http://www.pantherdb.org/tools/csnpScore.do) were used to analyze the effect of pheasant *PRNP* polymorphisms on pheasant PrP. The PROVEAN score was calculated as the pairwise alignment scores between the query protein sequences and the single locus variations of the other proteins (24). If the PROVEAN score is equal to or below −2.5, it is called a “deleterious” effect, and if it is higher than −2.5, it is called a “neutral” effect. AMYCO using the PAPA score and the pWALTZ score for calculation indicates the protein amyloid propensity (25). An AMYCO score below 0.45 indicates low amyloid propensity, and an AMYCO score above 0.78 indicates high amyloid propensity. PolyPhen-2 was used to predict the possible impact of an amino acid substitution on the structure and function of a protein using direct physical and comparative considerations. The PolyPhen-2 score was calculated from the position-specific independent count (PSIC) score of the wild-type and mutant amino acids (26). The score ranged from 0.0 to 1.0, and the results were divided into “benign,” “probably damaging” and “possibly damaging.” PANTHER measured the preservation time for PSEP (position-specific evolutionary preservation). PSEP was estimated to preserve a position in the current pheasant protein by tracing back to its reconstructed direct ancestors. A longer preservation time indicated that it had a deleterious effect (27). The score for preservation time was “Probably damaging [time>450 millions of years (my)],” “possibly damaging (450>time>200 my)” and “probably benign (time <200 my).”

### 3D Structure Modeling of Avian PrP

The 3D structure of avian PrPs was predicted by the SWISS-model program (https://swissmodel.expasy.org/) and IntFOLD program (https://www.Reading.ac.uk/bioinf/IntFOLD/). Homology-based modeling was performed by the SWISS-model program based on BLAST and HHblits from the SWISS-model template library (SMTL). The 3D structure of chicken PrP based on nuclear magnetic resonance spectroscopy (NMR) was obtained from a protein data bank (PDB ID:1U3M). The 3D structure of codons 95–230 of Pekin duck PrP was predicted by the SWISS-model program based on the NMR structure of human PrP (PDB ID:2lft.1.A). The 3D structure of codons 134–248 of wild type of pheasant PrP was predicted by the SWISS-model program based on the NMR structure of chicken PrP (PDB ID:1U3M). 3D modeling was performed using three criteria global model quality estimate (GMQE), qualitative model energy analysis (QMEAN) and sequence identity. IntFOLD is an integrated web resource for the prediction of the 3D structure of proteins. The prediction was performed by the ModFOLD6 server with intuitive local and global quality scores of 3D modeling. The 3D structure of codons 1–133 and 248–272 of pheasant PrP was predicted by the IntFOLD program. The predicted 3D structure of pheasant PrP was visualized by Swiss PDB viewer 4.1 software (Swiss Institute of Bioinformatics, Lausanne, Switzerland). The secondary structure and hydrogen bond of pheasant PrP were analyzed by PDB viewer 4.1 software. Hydrogen bonds were predicted if a hydrogen was in the range from 2.195 to 3.3 Å of an accessible donor atom.

## Results

### Identification of Novel Genetic Polymorphisms of the Pheasant *PRNP* Gene

**Figure 1:**
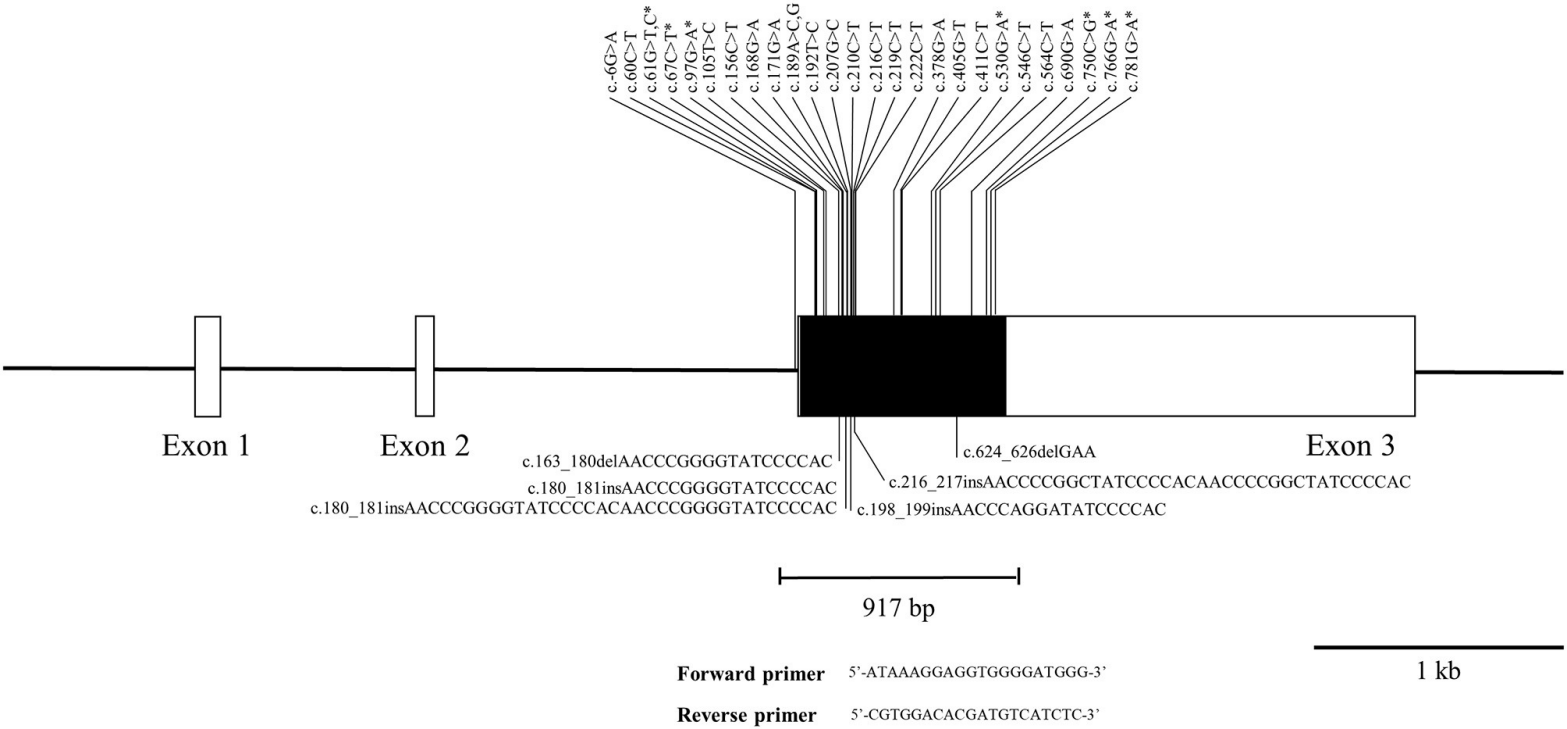
Novel genetic polymorphisms of the pheasant prion protein gene (*PRNP*) found in this study. The open reading frame (ORF) is indicated by a shaded block and the 5' and 3' untranslated regions (UTRs) are indicated by white blocks. The edged horizontal bar indicates the region sequenced. Asterisks denote nonsynonymous single nucleotide polymorphisms (SNPs). Upper lines indicate the novel SNPs. The lower lines indicate the novel insertion/deletion polymorphisms.

The pheasant *PRNP* gene is comprised of three exons and the ORF is located on exon 3. We amplified the pheasant *PRNP* gene using gene-specific primers, and the sequencing result was identical to the registered pheasant *PRNP* gene on GenBank (Gene ID: 116238382). To investigate polymorphisms of the pheasant *PRNP* gene, PCR and amplicon sequencing were performed in 148 pheasants. We found a total of 34 novel polymorphisms including 28 SNPs and 6 insertion/deletion polymorphisms (Figure 1, Supplementary Figures 1, 2). Of the 28 SNPs, 8 SNPs, c.61G>T (V21F), c.61G>C (V21L), c.67C>T (L23F), c.97G>A (G33C), c.530G>A (R177Q), c.750C>G (I250M), c.766G>A (D256N) and c.781G>A (V261I), were non-synonymous SNPs. We investigated the genotype and allele frequencies and HWE of the 34 polymorphisms of the pheasant *PRNP* gene (Table 1). Except for c.750C>G and c.766G>A, all genetic polymorphisms were in HWE. The genotype and allele frequencies of the 34 polymorphisms are described in Table 1. In addition, we analyzed LD among all polymorphisms of the pheasant *PRNP* gene with their D' and r^2^ value. Notably, all genetic polymorphisms have linkage disequilibrium (D' > 0). In addition, a total of 25 strong LDs (r^2^ > 0.333) were found (Table 2). Furthermore, we performed haplotype analysis of all polymorphisms of the pheasant *PRNP* gene and identified 14 major haplotypes (Table 3). Among the 14 haplotypes, the ACGCGTCWtGGInsWtATWtGCCWtCCGGCGCTWtACGA (13.1%) haplotype had the highest frequency, followed by the GCGCGTCWtGGWtWtATWtGCTWtCCGGTGTCWtGGAG (10.1%) and ACGCGTTWtGGWtWtATInsGCCInsCCGGCGCTWtGCGA (8.4%) haplotypes.

**Table 1:** Genotype and allele frequencies of pheasant prion protein gene (*PRNP*) polymorphisms in 148 pheasants.

**Polymorphisms**	**Genotype Frequency**, ***n*** **(%)**			**Allele Frequency**, ***n*** **(%)**		**HWE**
	**M/M**	**M/m**	**m/m**	**M**	**m**	
c.-6G>A	64 (43.24)	6 0(40.54)	24 (16.22)	188 (63.51)	108 (36.49)	0.127
c.60C>T	134 (90.54)	13 (8.78)	1 (0.68)	281 (94.93)	15 (5.07)	0.289
c.61G>T,C	GG 142 (95.95)	GT 5 (3.38)	TT 0 (0)	G 290 (97.97)	T 5 (1.69)	0.801
		GC 1 (0.68)	CC 0 (0)		C 1 (0.34)	
c.67C>T	138 (93.24)	9 (6.08)	1 (0.68)	285 (96.28)	11 (3.72)	0.068
c.97G>A	145 (97.97)	3 (2.03)	0 (0) 0	193 (98.99)	3 (1.01)	0.901
c.105T>C	133 (89.86)	14 (9.46)	1 (0.68)	280 (94.59)	16 (5.41)	0.362
c.156C>T	98 (66.22)	47 (31.76)	3 (2.03)	243 (82.09)	53 (17.91)	0.329
Ins/del type 1	147 (99.32)	1 (0.68)	0 (0)	295 (99.66)	1 (0.34)	0.967
c.168G>A	143 (96.62)	5 (3.38)	0 (0)	291 (98.31)	5 (1.69)	0.834
c.171G>A	143 (96.62)	5 (3.38)	0 (0)	291 (98.31)	5 (1.69)	0.834
Ins/del type 2	77 (52.03)	57 (38.51)	14 (9.46)	211 (71.28)	85 (28.72)	0.471
Ins/del type 3	147 (99.32)	1 (0.68)	0 (0)	295 (99.66)	1 (0.34)	0.967
c.189A>G,C	AA 118 (79.73)	AG 12 (8.11)	GG 1 (0.68)	A 264 (89.19)	G 14 (4.73)	0.818
		AC 16 (10.81)	CC 1 (0.68)		C 18 (6.08)	
c.192T>C	135 (91.22)	12 (8.11)	1 (0.68)	282 (95.27)	14 (4.73)	0.222
Ins/del type 4	115 (77.70)	32 (21.62)	1 (0.68)	262 (88.51)	34 (11.49)	0.441
c.207G>C	129 (87.16)	18 (12.16)	1 (0.68)	276 (93.24)	20 (6.76)	0.672
c.210C>T	120 (81.08)	24 (16.22)	4 (2.70)	264 (89.19)	32 (10.81)	0.053
c.216C>T	60 (40.54)	61 (41.22)	27 (18.24)	181 (61.15)	115 (38.85)	0.107
Ins/del type 5	115 (77.70)	32 (21.62)	1 (0.68)	262 (88.51)	34 (11.49)	0.441
c.219C>T	122 (82.43)	25 (16.89)	1 (0.68)	269 (90.88)	27 (9.12)	0.819
c.222C>T	143 (96.62)	5 (3.38)	0 (0)	291 (98.31)	5 (1.69)	0.834
c.378G>A	140 (94.59)	8 (5.41)	0 (0)	288 (97.30)	8 (2.70)	0.735
c.405G>T	140 (94.59)	8 (5.41)	0 (0)	288 (97.30)	8 (2.70)	0.735
c.411C>T	77 (52.03)	59 (39.86)	12 (8.11)	213 (71.96)	83 (28.04)	0.882
c.530G>A	124 (83.78)	24 (16.22)	0 (0)	272 (91.89)	24 (8.11)	0.283
c.546C>T	73 (49.32)	59 (39.86)	16 (10.81)	205 (69.26)	91 (30.74)	0.437
c.564C>T	55 (37.16)	71 (47.97)	22 (14.86)	181 (61.15)	115 (38.85)	0.906
Ins/del type 6	144 (97.30)	4 (2.70)	0 (0)	292 (98.65)	4 (1.35)	0.868
c.690G>A	63 (42.57)	68 (45.95)	17 (11.49)	194 (65.54)	102 (34.46)	0.834
c.750C>G	84 (56.76)	42 (28.38)	22 (14.86)	210 (70.95)	86 (29.05)	0.000
c.766G>A	105 (70.95)	33 (22.30)	10 (6.76)	243 (82.09)	53 (17.91)	0.003
c.781G>A	72 (48.65)	57 (38.51)	19 (12.84)	201 (67.91)	95 (32.09)	0.157

*M/M, Major homozygote; M/m, Heterozygote; m/m, Minor homozygote; M, Major allele; m, Minor allele; HWE, Hardy-Weinberg equilibrium, Ins/del Type 1: c.163_180delAACCCGGGGTATCCCCAC, Ins/del Type 2: c.180_181insAACCCGGGGTATCCCCAC, Ins/del Type 3: c.180_181insAACCCGGGGTATCCCCACA-ACCCGGGGTATCCCCAC, Ins/del Type 4: c.198_199insAACCCAGGATATCCCCAC, Ins/del Type 5: c.216_217insAACCCGGCTATCCCCACAACCCCGGCTATCCCCAC, Ins/del Type 6: c.624_626delGAA*.

**Table 2:** Linkage disequilibrium (LD) among genetic polymorphisms of the pheasant *PRNP* gene in pheasants.

	**1**	**2**	**3**	**4**	**5**	**6**	**7**	**8**	**9**	**10**	**11**	**12**	**13**	**14**	**15**	**16**	**17**	**18**	**19**	**20**	**21**	**22**	**23**	**24**	**25**	**26**	**27**	**28**	**29**	**30**	**31**	**32**
**1**	–	1	0.317	1	1	1	0.4	1	1	1	0.339	1	0.167	0.854	0.878	1	0.447	0.82	0.878	0.776	1	0.083	1	0.331	1	0.445	0.559	1	0.284	0.875	0.916	0.69
**2**	0.031	–	1	1	1	1	0.907	1	1	1	0.606	1	1	0.662	1	1	1	0.858	1	0.023	1	0.078	1	0.651	0.048	1	1	1	0.71	0.072	1	1
**3**	0.004	0.001	–	1	1	1	0.183	1	1	1	0.105	1	0.153	0.081	0.138	0.037	0.537	0.227	0.138	0.188	1	1	1	1	0.208	1	0.487	1	0.226	1	1	0.285
**4**	0.022	0.002	0.001	–	1	1	0.655	1	1	1	0.795	1	1	1	0.059	1	1	1	0.059	1	1	1	1	1	1	1	1	1	1	1	1	1
**5**	0.018	0.001	0	0	–	1	1	1	1	1	1	1	0.578	1	1	0.617	0.578	0.837	1	1	1	1	1	1	0.605	1	1	1	1	1	1	1
**6**	0.033	0.003	0.001	**0.675**	0.179	–	0.237	1	1	1	1	1	1	1	0.459	0.174	1	1	0.459	0.189	1	0.587	1	1	0.542	1	0.12	1	0.302	1	1	0.561
**7**	0.061	0.201	0	0.004	0.047	0.015	–	1	1	1	1	1	1	1	1	1	0.416	0.021	1	0.068	1	0.818	1	1	0.207	1	1	1	0.723	0.604	1	0.404
**8**	0.006	0	0	0	0	0	0.001	–	1	1	1	1	1	1	1	1	1	1	1	1	1	1	1	1	1	1	1	1	1	1	1	1
**9**	0.03	0.001	0	0.001	**0.596**	0.301	0.079	0	–	1	1	1	0.238	1	1	0.31	0.238	1	1	1	1	1	1	1	0.288	1	1	1	1	1	1	1
**10**	0.03	0.001	0	0.001	**0.596**	0.301	0.079	0	1	–	1	1	0.238	1	1	0.31	0.238	1	1	1	1	1	1	1	0.288	1	1	1	1	1	1	1
**11**	0.081	0.008	0.001	0.01	0.004	0.023	0.088	0.001	0.007	0.007	–	1	0.779	0.435	1	1	0.71	1	1	1	1	1	0.126	0.856	1	0.328	0.54	1	0.299	0.238	0.109	0.42
**12**	0.006	0	0	0	0	0	0.001	0	0	0	0.001	–	1	1	1	1	1	1	1	1	1	1	1	1	1	1	1	1	1	1	1	1
**13**	0.006	0.006	0.004	0.005	0.028	0.007	0.026	0.028	0.008	0.008	0.03	0	–	1	1	0.823	0.523	0.316	1	0.378	0.238	0.045	0.694	0.344	0.594	0.114	1	1	0.247	0.626	1	1
**14**	0.063	0.001	0.003	0.002	0.001	0.003	0.011	0.068	0.001	0.001	0.004	0	**0.41**	–	0.937	1	1	0.693	0.937	1	1	1	0.731	0.324	1	0.86	1	1	0.47	0.568	1	1
**15**	0.174	0.007	0.003	0.001	0.001	0.002	**0.595**	0	0.002	0.002	0.052	0	0.016	0.006	–	1	0.086	0.482	1	1	1	1	1	1	1	1	1	1	1	1	1	1
**16**	0.042	0.004	0	0.003	0.054	0	0.016	0	0.023	0.023	0.029	0	**0.405**	0.004	0.009	–	1	1	1	0.155	0.31	0.137	1	1	0.113	1	1	1	0.278	0.513	1	1
**17**	0.014	0.006	0.001	0.005	0.028	0.007	0.005	0	0.008	0.008	0.025	0	0.273	0.006	0	0.598	–	0.521	0.086	0.062	0.238	0.208	0.116	0.17	0.048	0.012	0.59	1	0.194	0.284	0.417	0.625
**18**	0.246	0.062	0.001	0.025	0.005	0.036	0	0.005	0.011	0.011	0.256	0.005	0.019	0.015	0.019	0.114	0.052	–	0.482	1	1	0.077	1	0.444	1	0.306	0.564	1	0.188	0.507	0.36	0.731
**19**	0.174	0.007	0.003	0.001	0.001	0.002	**0.595**	0	0.002	0.002	0.052	0	0.016	0.006	1	0.009	0	0.019	–	1	1	1	1	1	1	1	1	1	1	1	1	1
**20**	0.035	0	0.007	0.004	0.102	0.02	0.002	0	0.171	0.171	0.04	0	0.002	0.005	0.013	0.017	0.003	0.064	0.013	–	1	0.558	1	1	0.953	1	0.531	0.084	0.082	1	1	1
**21**	0.03	0.001	0	0.001	**0.596**	0.301	0.079	0	1	1	0.007	0	0.008	0.001	0.002	0.023	0.008	0.011	0.002	0.171	–	1	1	1	0.288	1	1	1	1	1	1	1
**22**	0	0.003	0.001	0.001	**0.369**	0.167	0.085	0	**0.619**	**0.619**	0.011	0	0	0.001	0.004	0.007	0.01	0	0.004	0.086	0.619	–	1	1	0.108	1	0.651	1	1	1	1	1
**23**	0.048	0.001	0.001	0.001	0	0.002	0.006	0.122	0	0	0.001	0	0.11	0.299	0.004	0.002	0.003	0.018	0.004	0.003	0	0.001	–	0.728	1	0.746	1	0.21	0.672	1	1	0.109
**24**	0.025	0.009	0.008	0.015	0.004	0.022	0.085	0.009	0.007	0.007	0.115	0.009	0.006	0.013	0.051	0.028	0.001	0.121	0.051	0.039	0.007	0.011	0.038	–	1	0.698	0.954	0.369	0.548	0.561	0.481	1
**25**	0.051	0.001	0.01	0.003	0.042	0.001	0.001	0	0.016	0.016	0.036	0	0.004	0.004	0.011	0.011	0.002	0.056	0.011	**0.799**	0.016	0.004	0.002	0.034	–	1	1	0.106	0.221	1	1	1
**26**	0.051	0.024	0.009	0.017	0.005	0.025	0.097	0.008	0.008	0.008	0.019	0.002	0.004	0.083	0.058	0.032	0	0.065	0.058	0.045	0.008	0.012	0.035	**0.427**	0.039	–	0.918	0.706	0.514	0.807	0.969	1
**27**	0.282	0.084	0.008	0.025	0.016	0.001	**0.343**	0.002	0.027	0.027	0.185	0.002	0.077	0.032	0.204	0.046	0.027	0.128	0.204	0.018	0.027	0.019	0.018	0.225	0.056	0.238	–	1	0.165	0.719	0.908	0.749
**28**	0.008	0.001	0	0.001	0	0.001	0.003	0	0	0	0.006	0	0.002	0.001	0.002	0.001	0.002	0.022	0.002	0.001	0	0	0.022	0.001	0.002	0.003	0.009	–	1	0.492	0.225	1
**29**	0.074	0.014	0.002	0.02	0.019	0.003	0.06	0.006	0.033	0.033	0.068	0.006	0.014	0.021	0.068	0.011	0.009	0.012	0.068	0.001	0.033	0.053	0.024	0.062	0.002	0.062	0.023	0.026	–	0.626	0.909	0.191
**30**	0.18	0.001	0.008	0.016	0.004	0.023	0.033	0.001	0.007	0.007	0.009	0.001	0.019	0.007	0.053	0.008	0.004	0.166	0.053	0.041	0.007	0.011	0.011	0.299	0.036	**0.601**	0.135	0.001	0.084	–	0.971	0.903
**31**	0.105	0.012	0.005	0.008	0.002	0.012	0.048	0.001	0.004	0.004	0.006	0.001	0.026	0.011	0.028	0.016	0.005	0.045	0.028	0.022	0.004	0.006	0.006	0.129	0.019	**0.462**	0.114	0.003	0.095	0.502	–	1
**32**	**0.392**	0.025	0.004	0.082	0.005	0.038	0.075	0.002	0.008	0.008	0.15	0.002	0.057	0.023	0.275	0.034	0.022	0.161	0.275	0.047	0.008	0.013	0	0.184	0.042	0.021	**0.417**	0.006	0.033	0.158	0.103	–

*The above diagonal and below diagonal indicate the D' value and r^2^ value, respectively. The value of the strong LD (r^2^ > 0.333) is emphasized in bold*.

*1:c.-6G>A; 2:c.60C>T; 3:c.61G>T,C; 4:c.67C>T; 5:c.97G>A; 6:c.105T>C; 7:c.156C>T; 8:c.163_180delAACCCGGGGTATCCCCAC; 9:c.168G>A; 10:c.171G>A; 11:c.180_181insAACCCGGGGTATCCCCAC; 12:c.180_181insAACCCGGGGTATCCCCACAACCCGGGGTATCCCCAC; 13:c.189A>G,C; 14:c.192T>C; 15:c.198_199insAACCCAGGATATCCCCAC; 16:c.207G>C; 17:c.210C>T; 18:c.216C>T; 19:c.216_217insAACCCGGCTATCCCCACAACCCCGGCTATCCCCAC; 20:c.219C>T; 21:c.222C>T; 22:c.378G>A; 23:c.405G>T; 24:c.411C>T; 25:c.530G>A; 26:c.546C>T; 27:c.564C>T; 28:c.624_626delGAA; 29:c.690G>A; 30:c.750C>G; 31:c.766G>A; 32:c.781G>A*.

**Table 3:** Haplotype frequency of 34 *PRNP* polymorphisms in pheasants.

	**c.-6G>A**	**c.60C>T**	**c.61G>T,C**	**c.67C>T**	**c.97G>A**	**c.105T>C**	**c.156C>T**	**c.163_180delAACCCGG** **GGTATCCCCAC**	**c.168G>A**	**c.171G>A**	**c.180_181insAACCCGG** **GGTATCCCCAC**	**c.180_181insAACCCGGGG** **TATCCCCACAACCCGGG** **GTATCCCCAC**	**c.189A>C,G**	**c.192T>C**	**c.198_199insAACCCAG** **GATATCCCCAC**	**c.207G>C**	**c.210C>T**	**c.216C>T**	**c.216_217insAACCCGGCT** **ATCCCCACAACCCCGGC** **TATCCCCAC**	**c.219C>T**	**c.222C>T**	**c.378G>A**	**c.405G>T**	**c.411C>T**	**c.530G>A**	**c.546C>T**	**c.564C>T**	**c.624_626delGAA**	**c.690G>A**	**c.750C>G**	**c.766G>A**	**c.781G>A**	**Frequency (n=148)**
**ht1**	A	C	G	C	G	T	C	WT	G	G	INS	WT	A	T	WT	G	C	C	WT	C	C	G	G	C	G	C	T	WT	A	C	G	A	39 (0.131)
**ht2**	G	C	G	C	G	T	C	WT	G	G	WT	WT	A	T	WT	G	C	T	WT	C	C	G	G	T	G	T	C	WT	G	G	A	G	30 (0.101)
**ht3**	A	C	G	C	G	T	T	WT	G	G	WT	WT	A	T	INS	G	C	C	INS	C	C	G	G	C	G	C	T	WT	G	C	G	A	25 (0.084)
**ht4**	G	C	G	C	G	T	C	WT	G	G	WT	WT	A	T	WT	G	C	C	WT	T	C	G	G	C	A	C	C	WT	G	C	G	G	18 (0.061)
**ht5**	G	C	G	C	G	T	C	WT	G	G	WT	WT	A	T	WT	G	C	T	WT	C	C	G	G	T	G	T	C	WT	G	G	G	G	18 (0.061)
**ht6**	G	C	G	C	G	T	C	WT	G	G	WT	WT	A	T	WT	G	C	T	WT	C	C	G	G	C	G	C	C	WT	A	C	G	G	13 (0.044)
**ht7**	G	C	G	C	G	T	C	WT	G	G	INS	WT	A	T	WT	G	C	C	WT	C	C	G	G	C	G	T	C	WT	G	G	A	G	12 (0.041)
**ht8**	G	C	G	T	G	C	C	WT	G	G	WT	WT	A	T	WT	G	C	C	WT	C	C	G	G	C	G	C	C	WT	G	C	G	A	11 (0.037)
**ht9**	G	C	G	C	G	T	C	WT	G	G	WT	WT	S	T	WT	C	T	T	WT	C	C	G	G	C	G	C	C	WT	G	C	G	G	10 (0.034)
**ht10**	G	C	G	C	G	T	C	WT	G	G	INS	WT	A	T	WT	G	C	C	WT	C	C	G	G	C	G	C	T	WT	G	C	G	G	9 (0.030)
**ht11**	G	T	G	C	G	T	T	WT	G	G	WT	WT	A	T	WT	G	C	T	WT	C	C	G	G	C	G	C	T	WT	G	C	G	G	7 (0.024)
**ht12**	A	C	G	C	G	T	C	WT	G	G	WT	WT	A	T	WT	G	C	C	WT	C	C	G	G	T	G	C	C	WT	G	C	G	G	5 (0.017)
**ht13**	A	C	G	C	G	T	T	WT	G	G	WT	WT	A	T	INS	G	C	T	INS	C	C	G	G	C	G	C	T	WT	G	C	G	A	5 (0.017)
**ht14**	A	C	G	C	G	T	C	WT	G	G	WT	WT	S	C	WT	G	C	C	WT	C	C	G	T	T	G	T	C	WT	G	C	G	G	5 (0.017)
Others^*^	-	-	-	-	-	-	-	-	-	-	-	-	-	-	-	-	-	-	-	-	-	-	-	-	-	-	-	-	-	-	-	-	89 (0.301)

* *Others contain rare haplotypes with frequency < 0.015. ‘S' indicates the nucleotide symbol of “Guanine/Cytosine”*.

### *In silico* Analysis of the Effect of Polymorphisms in the Pheasant *PRNP* Gene

We evaluated the functional and structural effects of *PRNP* polymorphisms on pheasant PrP using PolyPhen-2, PANTHER and PROVEAN (Table 4). Among the 8 non-synonymous SNPs, 5 non-synonymous SNPs including V21F, V21L, I250M, D256N and V261I were predicted to have benign effects on pheasant PrP in the PANTHER and PROVEAN programs. The L23F SNP was predicted to be “Neutral” and “Probably Damaging” by PROVEAN and PANTHER, respectively. G33C was predicted to be “Deleterious” and “Probably benign” by PROVEAN and PANTHER, respectively. In addition, R177Q was predicted to be “Neutral,” “Probably benign” and “Possibly Damaging” by PROVEAN, PANTHER and PolyPhen-2, respectively. Notably, 4 insertion/deletion polymorphisms, c.180_181insAACCCGGGGTATCCCCAC, c.180_181insAACCCGGGGTATCCCCACAACCCGGGGTATCCCCAC, c.198_199insAACCCAGGATATCCCCAC, c.216_217insAACCCCGGCTATCCCCACAACCCCGGCTATCCCCAC and c.624_626delGAA were predicted to have benign effects on pheasant PrP by PROVEAN. Notably, c.163_180delAACCCGGGGTATCCCCAC was predicted to be “Deleterious” by PROVEAN. We evaluated the amyloid propensity of pheasant PrP according to the alleles of the *PRNP* polymorphisms by AMYCO (Table 4). All polymorphisms were predicted to show a low amyloid propensity. However, R177Q showed an increase in AMYCO score (0.23) compared to wild type of pheasant PrP (0).

**Table 4:** *In silico* evaluation of the effect of *PRNP* polymorphisms of pheasant prion protein (PrP).

**Polymorphisms**	**PANTHER**		**PROVEAN**		**PolyPhen-2**		**AMYCO**
	**Score**	**Prediction**	**Score**	**Prediction**	**Score**	**Prediction**	**Score**
c.61G>T V21F	2	Probably benign	−1.160	Neutral		N.A.	0
c.61G>C V21L	2	Probably benign	−0.818	Neutral		N.A.	0
c.67C>T L23F	324	Probably Damaging	−0.653	Neutral		N.A.	0
c.97G>A G33C	2	Probably benign	−2.840	Deleterious		N.A.	0
c.163_180delAACCCGGGGTATCCCCAC		N.A.	−14.115	Deleterious		N.A.	0
c.180_181insAACCCGGGGTATCCCCAC		N.A.	12.740	Neutral		N.A.	0
c.180_181insAACCCGGGGTATCCCCAC AACCCGGGGTATCCCCAC		N.A.	23.091	Neutral		N.A.	0
c.198_199insAACCCAGGATATCCCCAC		N.A.	12.740	Neutral		N.A.	0
c.216_217insAACCCCGGCTATCCCCAC AACCCCGGCTATCCCCAC		N.A.	23.091	Neutral		N.A.	0
c.530G>A R177Q	2	Probably benign	−0.413	Neutral	0.907	Possibly Damaging	0.23
c.624_626delGAA		N.A.	−0.004	Neutral		N.A.	0
c.750C>G I250M	2	Probably benign	−0.188	Neutral	0.306	Benign	0
c.766G>A D256N	2	Probably benign	0.526	Neutral		N.A.	0
c.781G>A V261I	2	Probably benign	−0.081	Neutral		N.A.	0

*N.A., Not available*.

**Figure 2:**
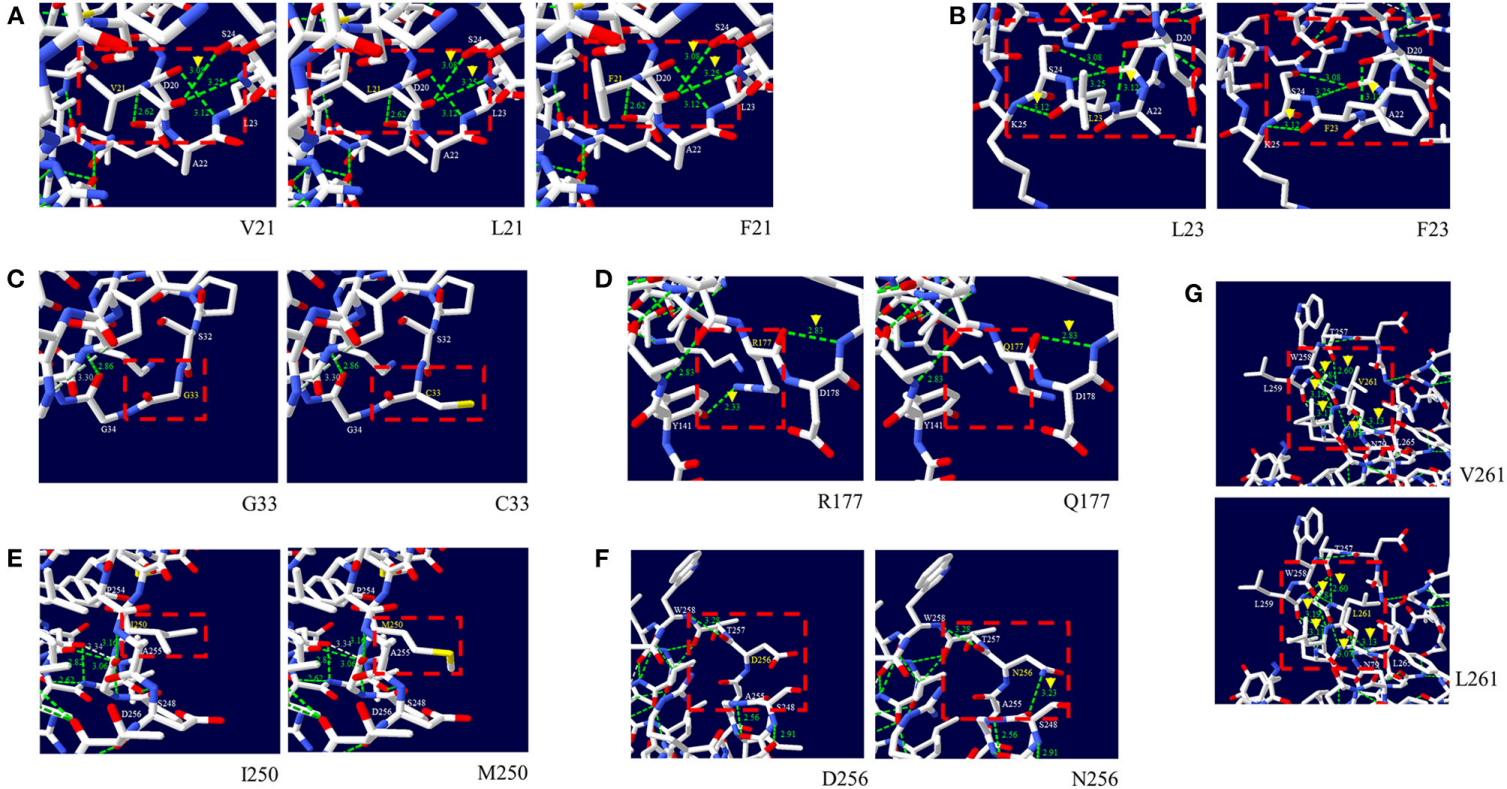
Prediction of the tertiary structure and hydrogen bonds of the pheasant prion protein (PrP) according to the alleles of the non-synonymous SNPs of the pheasant *PRNP* gene. **(A)** 3D structure of pheasant PrP with the V21, L21, and F21 alleles. **(B)** 3D structure of the pheasant PrP with the L23 and F23 alleles. **(C)** 3D structure of the pheasant PrP with the G33 and C33 alleles. **(D)** 3D structure of the pheasant PrP with the R177 and Q177 alleles. **(E)** 3D structure of the pheasant PrP with the I250 and M250 alleles. **(F)** 3D structure of the pheasant PrP with the D256 and N256 alleles. **(G)** 3D structure of the pheasant PrP with the V261 and L261 alleles. The green dotted line indicates hydrogen bonds. The green numbers indicate the distance of the hydrogen bonds.

Next, we analyzed the effect of nonsynonymous SNPs on the 3D structure of pheasant PrP (Figure 2). The V21, L21 and F21 alleles showed two hydrogen bonds with S24 (3.08 Å and 3.25 Å) (Figure 2A). The L23 and F23 alleles showed two hydrogen bonds, with K25 (3.12 Å) and with D20 (3.12 Å) (Figure 2B). The G33 and C33 alleles did not have hydrogen bonds (Figure 2C). The R177 allele showed two hydrogen bonds with Y141 (2.33 Å) and D178 (2.83 Å) (Figure 2D). Notably, the Q177 allele showed only one hydrogen bond with D178 (2.83 Å) (Figure 2D). The I250 and M250 alleles did not have hydrogen bonds (Figure 2E). The D256 allele did not have a hydrogen bond. However, the N256 allele had one hydrogen bond with S248 (3.23 Å) (Figure 2F). The V261 and L261 alleles showed 6 identical hydrogen bonds with N79 (3.07 Å), T257 (2.60 Å), W258 (2.84 Å and 3.19 Å), L259 (3.11 Å) and L265 (3.13 Å) (Figure 2G).

### Comparison of Tandem Repeat Domains of Pheasants and Several Species

**Figure 3:**
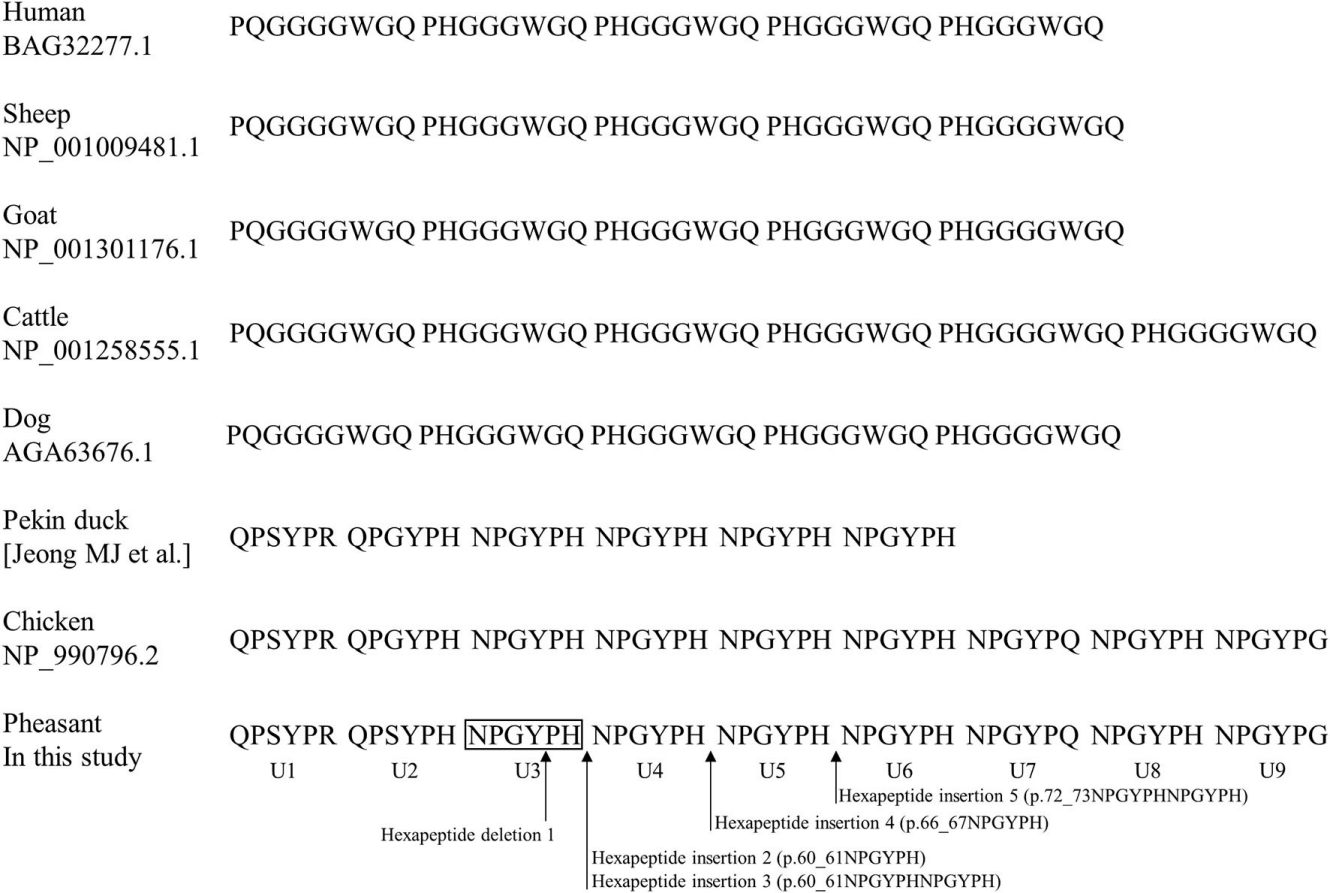
Comparison of tandem repeat sequences in humans, sheep, goats, cattle, dogs, Pekin ducks, chickens and pheasants. The amino acid sequences of the tandem repeat region were obtained from GenBank at the National Center for Biotechnology Information (NCBI), including human (BAG32277.1), sheep (NP_001009481.1), goat (NP_001301176.1), cattle (NP_001258555.1), dog (AGA63676.1), Pekin duck (21), chicken (NP_990796.2) and pheasant (In this study). The arrows indicate the insertion/deletion polymorphisms found in this study. U 1-9: hexapeptide repeat 1-9.

We compared amino acid sequences of tandem repeat domains among human, sheep, goat, cattle, dog, Pekin duck, chicken and pheasant PrPs (Figure 3). Mammalian and avian PrPs showed octapeptide repeat domains (PHGGGWGQ) and hexapeptide repeat domains (NPGYPH), respectively. Pheasant PrP has 9 units of hexapeptide repeat domains, which are identical in length to those of chickens. Except for the second tandem repeat unit (chicken: QPGYPH, pheasant: QPSYPH), the amino acid sequences of the tandem repeat domains of pheasant PrP are identical to those of chicken. However, Pekin duck PrP showed shorter tandem repeat domains than pheasant and chicken PrPs (Pekin duck: 36 aa, pheasant: 54 aa, chicken: 54 aa).

### Comparison of Secondary and Tertiary Structures of Pheasants and Avian PrPs

**Figure 4:**
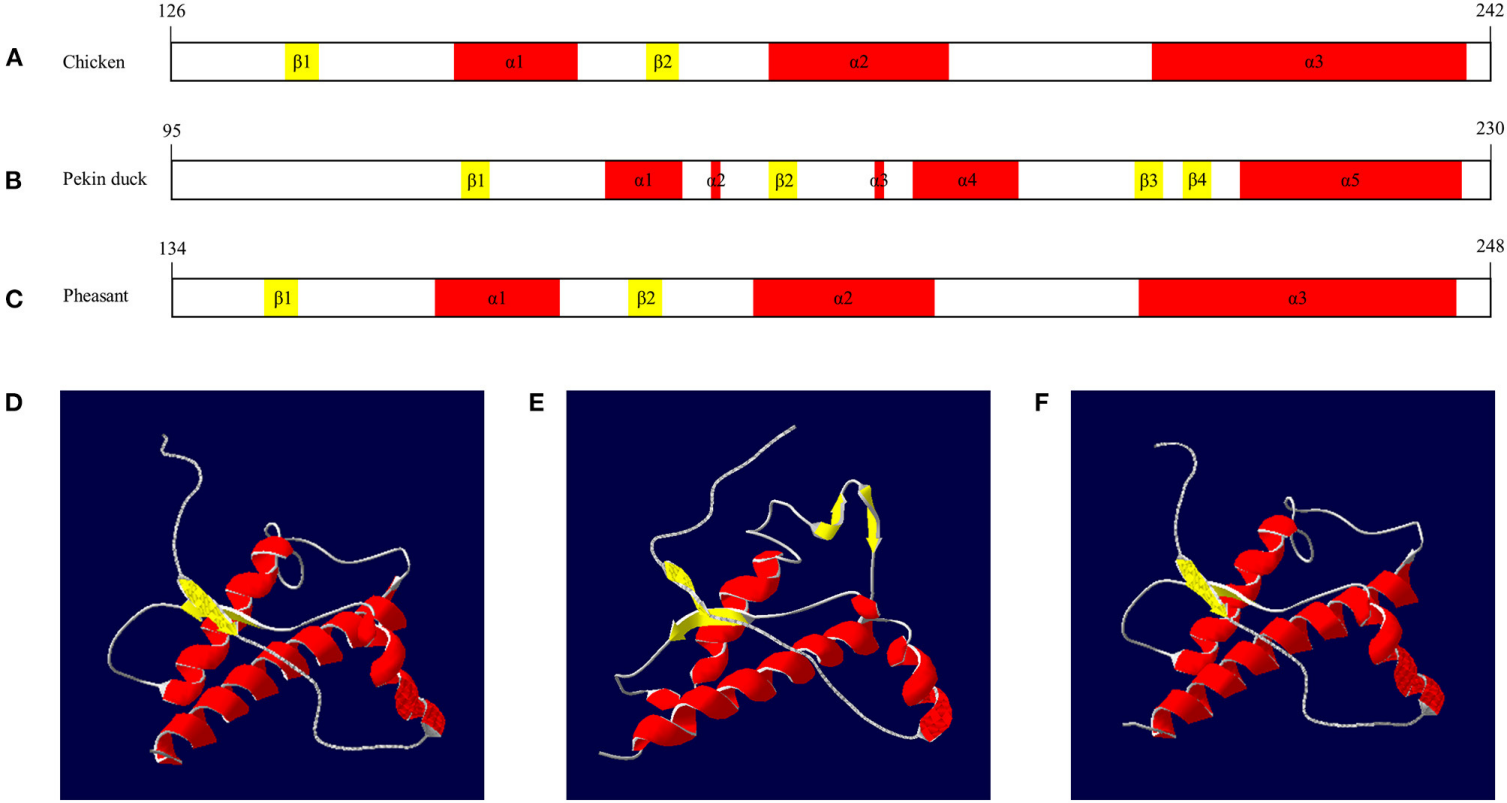
The secondary and tertiary structures of avian PrPs. **(A)** The secondary structure of the chicken PrP. **(B)** The secondary structure of the Pekin duck PrP. **(C)** The secondary structure of the pheasant PrP. **(D)** The tertiary structure of the chicken PrP. **(E)** The tertiary structure of the Pekin duck PrP. **(F)** The tertiary structure of the pheasant PrP. The colors indicate α-helices (red), β-sheets (yellow) and coils (white). The secondary and tertiary structures of avian PrPs were analyzed by SWISS-MODEL and Swiss PDB Viewer.

We compared the secondary and tertiary structures of avian PrPs by Swiss-model and Swiss PDB viewer. Chicken PrP was predicted to have two β-sheet structures (codons 136–138, 168–170) and three α-helix structures (codons 157–167, 185–200 and 219–246) (Figure 4A). Pekin duck PrP was predicted to have four β-sheet structures (codons 125–127, 157–159, 195–197 and 200–202) and five α-helix structures (140–147, 151, 168, 172–182 and 206–228) (Figure 4B). Pheasant PrP was predicted to have two β-sheet structures (codons 142–144 and 174–176) and three α-helix structures (codons 157–167, 185–200 and 219–246) (Figure 4C). The tertiary structures of chicken and pheasant PrPs showed three α-helices and two antiparallel β-sheet structures (Figures 4D,F) However, the tertiary structure of Pekin duck PrP showed five α-helices and four antiparallel β-sheet structures (β1-β2 and β3-β4) (Figure 4E).

## Discussion

In this study, we found a total of 34 novel polymorphisms of the pheasant *PRNP* gene including 8 non-synonymous SNPs and 6 insertion/deletion polymorphisms (Figure 1, Table 1). Except for c.750C>G (I250M) and c.766G>A (D256N), all genetic polymorphisms were in HWE (Table 1). Since c.750C>G (I250M) and c.766G>A (D256N) are non-synonymous SNPs, functional alteration caused by the non-synonymous SNPs may be related to genetic selection and induced the HWE violation. Among the 8 non-synonymous SNPs, 2 SNPs including G33C and R177Q were predicted to have deleterious effects on pheasant PrP by *in silico* analyses (Table 4). In addition, L23F was predicted to have damaging effect on pheasant PrP by PANTHER. Since the L23F and G33C SNPs, which are located in the interspecies-conserved signal peptide of PrP, were predicted to have a deleterious effect on pheasant PrP, further analysis of the signal peptide of pheasant PrP according to the allele of the pheasant *PRNP* gene is highly desirable. The R177Q SNP, which is located in the structured region of pheasant PrP, confers an increase in amyloid propensity (Table 4). Although R177Q has a higher AMYCO score than other polymorphisms, it does not exceed the threshold with a low amyloid propensity. It indicates that genetic polymorphisms of the pheasant *PRNP* gene may be induced low amyloid propensity and pheasant PrP has a relatively stable protein structure. In addition, pheasant PrP with Q177 showed a reduction in the number of hydrogen bonds compared to pheasant PrP with R177. Since hydrogen bonds play a pivotal role in the stability of PrP and the stability of PrP confers resistance to the conformational changes in PrP (28), the R177Q SNP was thought to be a potential risk factor for prion diseases. Further investigation of the *in vitro* and/or *in vivo* evaluation of the amyloid propensity of pheasant PrP with the R177 allele and Q177 allele is needed in the future. However, although several genetic polymorphisms have strong genetic linkages, the effect of each SNP and insertion/deletion were only evaluated individually due to the internal setting of the software. Further evaluation of sequence variations of the pheasant *PRNP* gene according to haplotypes is highly desirable in the future.

We identified a highly polymorphic tandem repeat region of the pheasant *PRNP* gene (Figures 1, 3). The tandem repeat region of PrP is related to the activity of copper-related enzymes *via* the binding of copper ions in mammals (histidine) and birds (histidine, tyrosine) (29, 30). Since pheasant has additional copper binding sites, the insertion/deletion polymorphisms of the pheasant *PRNP* gene would not cause critical dysfunction of the copper-related activity of pheasant PrP and genetic polymorphisms of the pheasant tandem repeat region seem to be more frequently observed than in mammals. Previous studies have reported that insertion/deletion polymorphisms in the octapeptide repeat region of the *PRNP* gene are associated with vulnerability to CJD (31). Since insertion/deletion polymorphisms are a potent risk factor for prion disease, further analysis on relationship between insertion/deletion polymorphisms of the pheasant *PRNP* gene and susceptibility to prion disease is needed in the future.

We also found that the secondary and tertiary structures of the pheasant PrP have two β-sheets and three α-helices, similar to chicken PrP. Conversely, the pheasant PrP showed different secondary and tertiary structures from those of Pekin duck, which has 4 β-sheets and 5 α-helices. Since chickens showed resistance to BSE infection and pheasant PrP has a PrP structure similar to that of chicken PrP, pheasant PrP is predicted to have a relatively prion-resistant structure. Further validation is needed in the future using transgenic mice carrying pheasant PrP.

## Conclusion

In the present study, we identified 34 novel genetic polymorphisms of the pheasant *PRNP* gene including 8 non-synonymous SNPs and 6 insertion/deletion polymorphisms in 148 pheasants. Among the 8 non-synonymous SNPs, the L23F, G33C and R177Q SNPs were predicted to have a deleterious effect on pheasant PrP. In addition, the R177Q SNP induced an increase in amyloid propensity and a reduction in hydrogen bonds. Among the 6 insertion/deletion polymorphisms, the c.163_180delAACCCGGGGTATCCCCAC polymorphism was predicted to have a deleterious effect on pheasant PrP. Furthermore, the secondary and tertiary structures of the pheasant PrP are very similar to those of chicken PrP. To the best of our knowledge, this is the first report on genetic polymorphisms of the pheasant *PRNP* gene.

## Data Availability Statement

Data are available on reasonable request. Requests may be made to bhjeong@jbnu.ac.kr.

## Ethics Statement

The animal study was reviewed and approved by Institutional Animal Care and Use Committee (IACUC) of Jeonbuk National University.

## Author Contributions

KK, Y-CK, and B-HJ conceived, designed the experiment, analyzed the data, and wrote the paper. KK and Y-CK performed the experiments. All authors read and approved the final manuscript.

## Funding

KK was supported by the BK21 Plus Program in the Department of Bioactive Material Sciences. This research was supported by the Basic Science Research Program through the National Research Foundation (NRF) of Korea funded by the Ministry of Education (2017R1A6A1A03015876, 2021R1A6A3A010864). This work was supported by the National Research Foundation of Korea (NRF) grant funded by the Korean government (MSIT) (2021R1A2C1013213, 2022R1C1C2004792) (NRF-2019-Fostering Core Leaders of the Future Basic Science Program/Global Ph.D. Fellowship Program).

## Conflict of Interest

The authors declare that the research was conducted in the absence of any commercial or financial relationships that could be construed as a potential conflict of interest.

## Publisher's Note

All claims expressed in this article are solely those of the authors and do not necessarily represent those of their affiliated organizations, or those of the publisher, the editors and the reviewers. Any product that may be evaluated in this article, or claim that may be made by its manufacturer, is not guaranteed or endorsed by the publisher.

## Supplementary Material

Supplementary Figure 1 Electropherograms of 28 novel single nucleotide polymorphisms (SNPs) of the *PRNP* gene in pheasants. Four colors indicate the individual bases of the DNA sequence using an ABI3730xl automatic sequencer (blue: cytosine, red: thymine, black: guanine, green: adenine). The upper panels indicate major homozygotes, the middle panels indicate heterozygotes, and the lower panels indicate minor homozygotes.

Supplementary Figure 2 Electropherograms of the insertion/deletion polymorphisms of the pheasant *PRNP* gene. **(A)** c.163_180delAACCCGGGGTATCCCCAC (p.55_60delNPGYPH) **(B)** c.180_181insAACCCGGGGTATCCCCAC (p.60_61insNPGYPH) **(C)** c.180_181insAACCCGGGGTATCCCCACAACCCGGGGTATCCCCAC (p.60_61insNPGYPHNPGYPH) **(D)** c.198_199insAACCCAGGATATCCCCAC (p.66_67insNPGYPH) **(E)** c.216_217insAACCCCGGCTATCCCCACAACCCCGGCTATCCCCAC (p.72_73insNPGYPHNPGYPH) **(F)** c.624_626delGAA (p.208delK). Four colors indicate individual bases of the DNA sequence (blue: cytosine, red: thymine, black: guanine, green: adenine). WT: wild type of the pheasant prion protein (*PRNP*) gene.
