# Supplementary figures and images for: Novel Polymorphisms and Genetic Characteristics of the Prion Protein Gene in Pheasants
Source: Front Vet Sci. 2022 Jul 12;9:935476. doi: 10.3389/fvets.2022.935476 (PMC9322948; doi:10.3389/fvets.2022.935476)

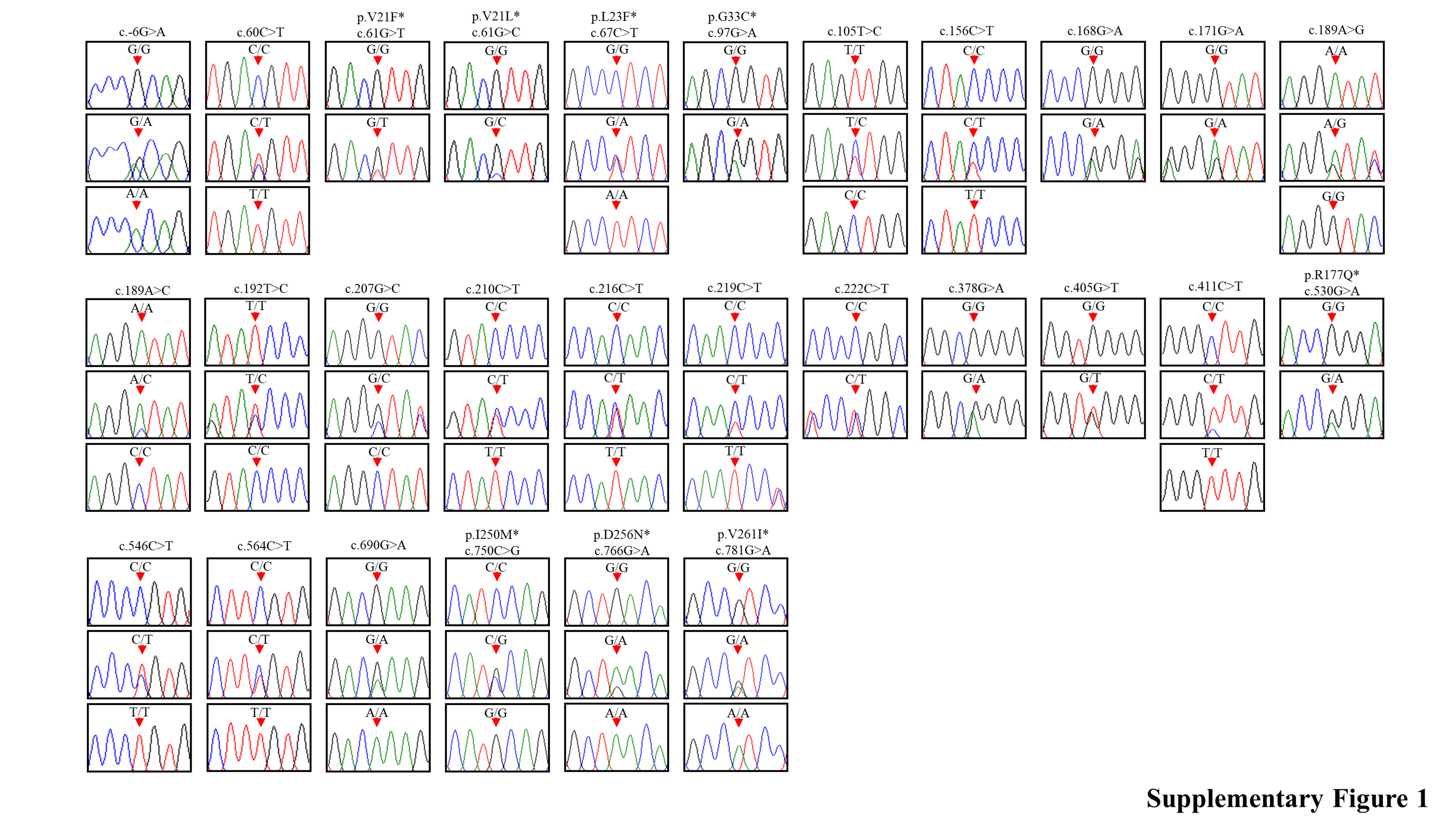

Supplement: Supplementary Figure 1 — Electropherograms of 28 novel single nucleotide polymorphisms (SNPs) of the PRNP gene in pheasants. Four colors indicate the individual bases of the DNA sequence using an ABI3730xl automatic sequencer (blue: cytosine, red: thymine, black: guanine, green: adenine). The upper panels indicate major homozygotes, the middle panels indicate heterozygotes, and the lower panels indicate minor homozygotes. [file Image_1.TIF]

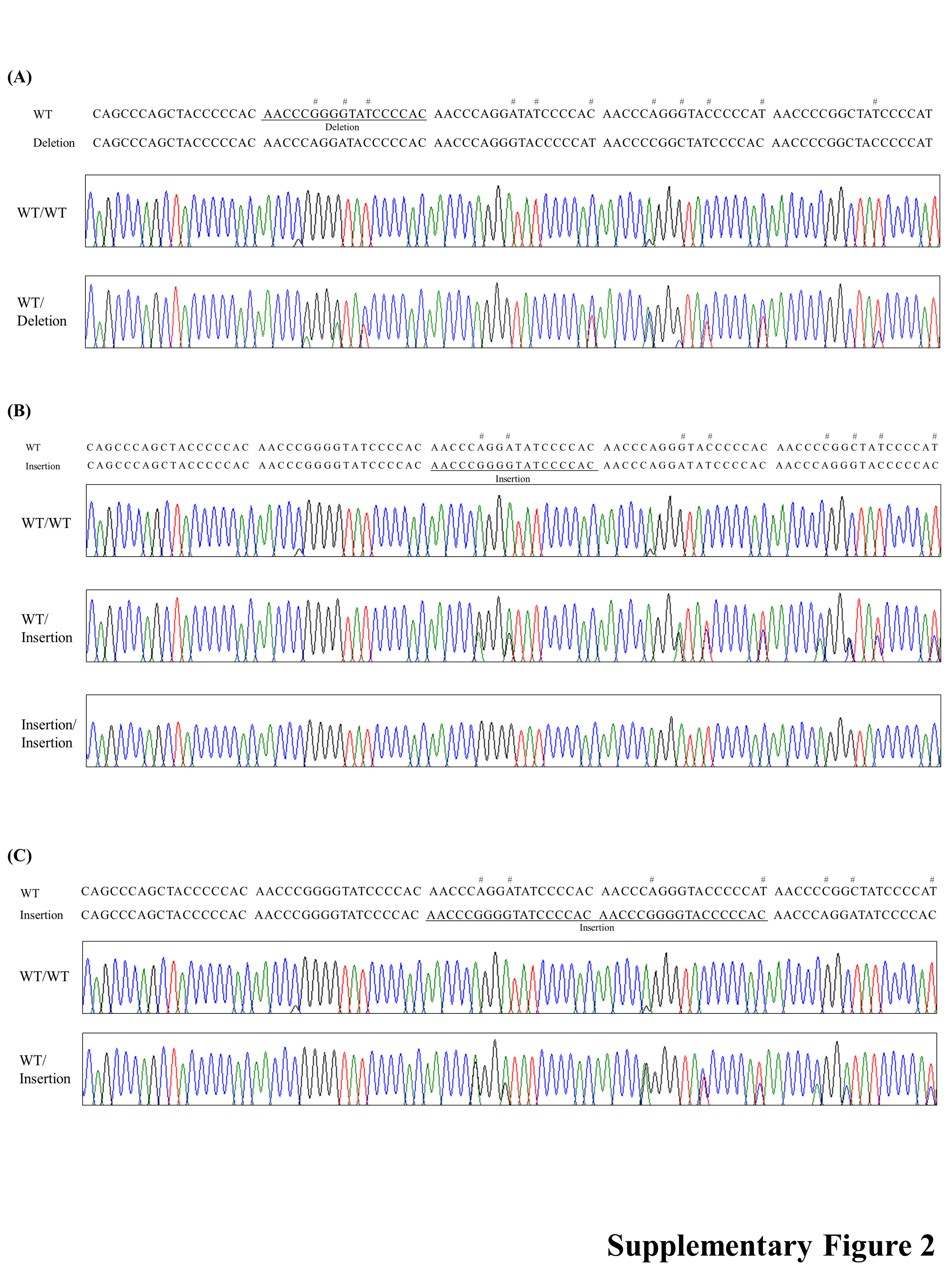

Supplement: Supplementary Figure 2 — Electropherograms of the insertion/deletion polymorphisms of the pheasant PRNP gene. (A) c.163_180delAACCCGGGGTATCCCCAC (p.55_60delNPGYPH) (B) c.180_181insAACCCGGGGTATCCCCAC (p.60_61insNPGYPH) (C) c.180_181insAACCCGGGGTATCCCCACAACCCGGGGTATCCCCAC (p.60_61insNPGYPHNPGYPH) (D) c.198_199insAACCCAGGATATCCCCAC (p.66_67insNPGYPH) (E) c.216_217insAACCCCGGCTATCCCCACAACCCCGGCTATCCCCAC (p.72_73insNPGYPHNPGYPH) (F) c.624_626delGAA (p.208delK). Four colors indicate individual bases of the DNA sequence (blue: cytosine, red: thymine, black: guanine, green: adenine). WT: wild type of the pheasant prion protein (PRNP) gene. [file Image_2.TIF]

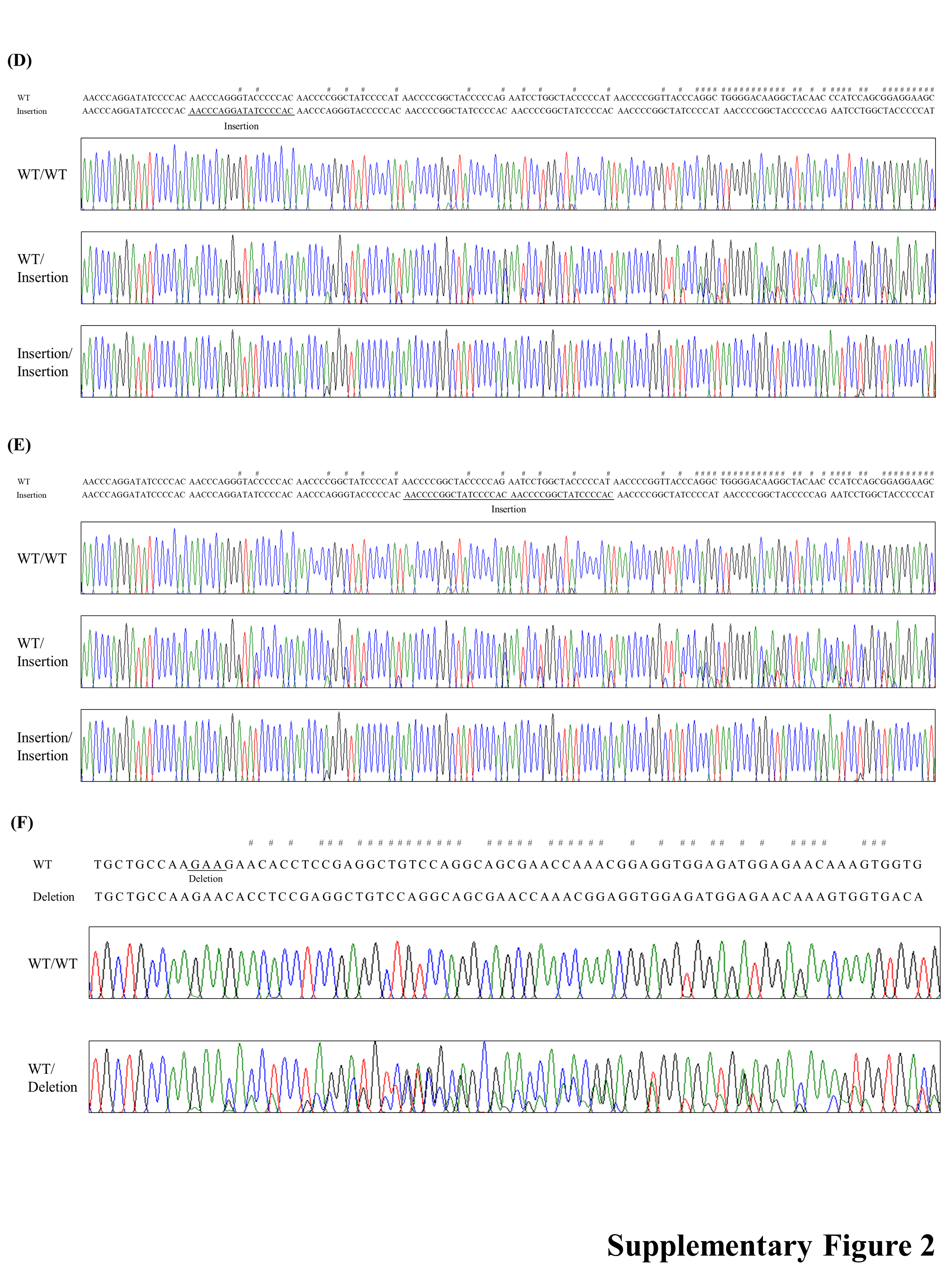

Supplement: Supplementary file 3 [file Image_3.TIF]
